# Functional and Emotional Impact of Dry Eye and Meibomian Gland Dysfunction in Keratoconus

**DOI:** 10.3390/biomedicines13081918

**Published:** 2025-08-06

**Authors:** Liat Gantz, Avi Besser, Rivki Bloom, Reut Ifrah

**Affiliations:** 1 Department of Optometry and Vision Science, Jerusalem Multidisciplinary College, Jerusalem 91010, Israel; rivkibl@edu.hac.ac.il (R.B.); reutif@jmc.ac.il (R.I.); 2 Department of Communication disorders, Jerusalem Multidisciplinary College, Jerusalem 91010, Israel; avibe@jmc.ac.il

**Keywords:** keratoconus, dry eye, meibomian gland dysfunction (MGD), quality of life, KEPAQ (keratoconus end-points assessment questionnaire), emotional impact, functional impact

## Abstract

**Background/Objectives**: Dry eye (DE) can cause persistent eye rubbing, contributing to keratoconus (KC) development and progression. Both keratoconus (KC) and dry eye (DE) significantly impact patients’ functional and emotional well-being, with KC patients exhibiting a higher prevalence of DE symptoms and signs. This study examined whether functional (KEPAQ-F) and emotional (KEPAQ-E) quality of life, assessed by the Keratoconus End-Points Assessment Questionnaire, differ when influenced by symptoms and clinical signs of general DE versus meibomian gland dysfunction (MGD) in KC patients. **Methods**: Volunteers with KC (ages 18–70) underwent DE and MGD assessments, completing OSDI, MGD (MGDQ), and KEPAQ questionnaires. Clinical measures included NITBUT, Schirmer, and meibography. Pearson correlations and path analysis assessed relationships between DE and MGD symptoms and KEPAQ-F/E. **Results**: Forty-five KC participants (mean age: 45 ± 13, range: 20–69 years, 25 males) were enrolled; 22 (49%) had DE, and 15 (33%) had MGD. Significant correlations were observed between KEPAQ-E (2.9 ± 3.0 Logit) and KEPAQ-F (1.7 ± 3.0 Logit) scores with OSDI (26.5 ± 26.7) and MGDQ (3.3 ± 2.2) scores, and all Belin outcome measures A-D for all participants. In participants with diagnosed dry eye, KEPAQ E and F were also significantly correlated with loss of meibomian glands in the lower eyelids (R = −0.44, *p* = 0.04). Path analysis showed both DE and MGD were negatively correlated with lower KEPAQ-E and KEPAQ-F scores, with DE symptoms more influential (*p* < 0.05). The model explained 42% of the KEPAQ-E variance and 41% of the KEPAQ-F variance. **Conclusions**: Emotional and functional quality of life in KC is significantly and negatively related to DE and MGD symptoms, with DE symptoms exhibiting a greater impact. Furthermore, greater loss of meibomian glands in the lower eyelids is significantly associated with reduced emotional and functional KEPAQ scores in DE patients. These results underscore the critical importance of evaluating DE in KC to improve patient-reported outcomes.

## 1. Introduction

Dry eye (DE) is a multifactorial, symptomatic disease marked by a disruption of tear film homeostasis and/or ocular surface, in which tear film instability and hyperosmolarity, inflammation and damage to the ocular surface, and neurosensory abnormalities are etiological factors [1].

The prevalence of DE ranges from about 5% to 50% [2], with a higher occurrence in women and which increases with age [3]. Meibomian gland dysfunction (MGD) is a primary cause of evaporative dry eye [4], defined as a chronic and diffuse abnormality of the meibomian glands (MGs), characterized by obstruction of the terminal ducts and/or changes in the quality or quantity of gland secretions [5].

DE affects visual function, daily activities, work productivity, and overall quality of life [6,7]. Direct costs, including doctor visits, medications, and procedures, alongside indirect costs such as reduced productivity and diminished quality of life, contribute to a significant economic burden associated with DE disease [6,7,8]. DE can lead to persistent eye rubbing [9], which may play a role in the pathogenesis of keratoconus (KC) [10,11]. KC is a progressive and bilateral asymmetrical corneal ectasia, causing thinning of the para-central portion of the cornea, resulting in distorted and decreased vision [12,13,14,15]. KC onset varies from early teenage years to young adulthood and rarely occurs after age 35 [13]. The prevalence of KC in different regions of the world varies from 0.0003% in Russia to 2.3% in Maharashtra, India; in Jerusalem, Israel, the prevalence of KC was found to be 2.34% [16].

KC causes changes in the corneal surface that reduce tear film quality [17]. Individuals with KC have been reported to complain of more DE symptoms and demonstrate more signs of DE, such as lower tear film volume and greater corneal staining [18].

Despite the availability of various surgical interventions and optical aids aimed at improving visual quality for individuals with KC, many individuals with this condition face challenges in effectively performing daily tasks [19]. Ectatic disorders have been shown to significantly impact emotional well-being [20], with individuals with KC exhibiting a higher prevalence of clinical depression [21] and other mental health conditions [22] compared to the general population. The “Keratoconus End-Points Assessment Questionnaire” (KEPAQ) is a validated tool designed to assess both the functional and emotional impacts of KC [23]. Patient-reported outcome measures are increasingly recognized as essential tools for capturing the disease burden from the patient’s perspective [23,24].

The primary objective of this study was to determine whether the presence of dry eye (DE) or meibomian gland dysfunction (MGD) is associated with differences in emotional and functional quality of life in individuals with keratoconus. Specifically, we examined whether KEPAQ-E (emotional) and KEPAQ-F (functional) scores are differentially influenced by subjective symptoms and clinical signs of general DE versus those specific to MGD. Correlation and path analyses were applied to explore these associations while accounting for potential confounding effects related to keratoconus severity.

## 2. Methods

This cross-sectional prospective observational study conformed to the ethical principles of the Declaration of Helsinki and was approved by the internal ethics committee of Jerusalem Multidisciplinary College (JMC), Israel (Approval Number 0091, 6 February 2025). The methods were explained verbally, and participants signed a statement of informed consent prior to their participation.

### 2.1. Participants

This study included KC patients of any type of severity. Presence of dry eye or MGD was not an inclusion criterion. Patients with KC were only included if they were previously diagnosed by an ophthalmologist. Their KC diagnosis was verified by the experimenter using the Belin ABCD Classification [25]. Exclusion criteria comprised self-reported ongoing ocular surface disease, allergic, atopic, or vernal keratoconjunctivitis, use of systemic medications associated with dry eye or MGD (e.g., antihistamines, hormones), pregnancy, or inability to provide informed consent. Additionally, patients with systemic diseases (e.g., multiple sclerosis, myasthenia gravis), based on self-reported history were not included.

### 2.2. Procedures

Examinations were performed at the contact lens clinics of the Optometry Department at JMC in a designated examination room, by a certified optometrist. A comprehensive review of medical and ocular history, along with lifestyle factors, was conducted to verify eligibility for the study. Participants were instructed to refrain from contact lens use on the day of the study visit and to avoid using artificial tears for at least two hours prior to the study visit [26]. After measurement of habitually corrected distance visual acuity (LogMAR chart), diagnosis of KC was verified using corneal tomography (Sirius Scheimpflug Camera; Bon Optic VertriebsgmnH, Lübeck, Germany) and slit lamp biomicroscopy (Huvitz Slit Lamp HS-5000, Anyang, Republic of Korea). KC grading was determined using the Belin ABCD Classification, as it is an effective method for assessing both anatomical and functional impairment in KC [25,27]. This classification comprises four criteria evaluating different aspects of corneal structure and vision. “A” represents anterior radius of curvature in the 3.0 mm zone centered on the thinnest location of the cornea, while “B” refers to posterior radius of curvature in the same zone. “C” denotes the thinnest pachymetry in µm, and “D” corresponds to the distance best-corrected visual acuity. All criteria, except for “D”, were derived directly from the Sirius Scheimpflug Camera images.

Dry eye-related outcome measures included the following tests in the following order. The non-invasive tear-film break-up time (NITBUT, Sirius Scheimpflug Camera Bon Optic VertriebsgmnH, Lübeck, Germany) assessed the time, in seconds, between the full opening of the eyelids after blinking and the first break in the tear film. Three consecutive readings were measured, and the average was calculated [28]. Tear meniscus height, the most direct non-invasive method for assessing tear film volume, was measured using a slit lamp [28]. Corneal and conjunctival staining were graded according to the Oxford grading scale [29,30]. Meibography images were captured using the Cobra HD fundus camera meibographer (CSO, Firenze, Italy, csoitalia.it). This instrument was previously reported to provide reliable meibography measurements [31]. The degree of meibomian gland loss was automatically calculated with the Phoenix software integral to the device and expressed as a percentage [32]. Tear production was subsequently measured using the Schirmer I test without topical anesthesia. A standard paper strip was placed in the mid-lateral portion of the lower fornix with the patient’s eyes closed. The examiner recorded the amount of wetting after five minutes. A value greater than 10 mm was considered normal [33,34].

The meibomian gland secretion was obtained by applying pressure to the eight central glands in the lower tarsus and maintaining the pressure for 10 s, using the Meibomian Gland Evaluator (Tear Science, Inc., Morrisville, NC, USA), a handheld device for evaluation of MG secretion under slit lamp visualization [35]. This device provides a standardized applied pressure between 0.8–1.2 g/mm^2^ for 10–15 s and has been shown to be repeatable in applying consistent, and gentle pressure to the outer skin of the lower eyelid while visualizing the secretions from the MG orifice [36,37]. The meibomian quality score (MQS) was scored as follows: 0: no secretions (blocked or atrophied glands), 1: inspissated (paste-like or semisolid) secretions, 2: cloudy liquid secretions, and 3: clear liquid secretions, while the meibomian expressibility score (MES) was scored as follows: 0: no glands expressible, 1: 1–2 glands expressible, 2: 3–4 glands expressible, and 3: all glands expressible [35,37].

Diagnosis of DE was based on an OSDI score ≥ 13 and NITBUT < 10 s [38]. Participants with DE and meibomian gland loss > 25% were classified as having MGD [5]. Participants completed an online Google Form which included the Meibomian Gland Dysfunction Questionnaire (MGDQ) [39], the OSDI questionnaire to assess the severity of ocular surface symptoms [40], and the Keratoconus End-Points Assessment Questionnaire (KEPAQ) to measure functional and emotional compromise in KC.

The OSDI questionnaire consists of twelve items assessing the respondent’s experiences over the past week, focusing on ocular symptoms, vision-related function, and environmental triggers. The OSDI score is calculated using the following formula: (sum of scores for all questions answered) × 25/(total number of questions answered), which is scored on a scale of 0 to 100 [41]. A score less than 13 is considered normal [38]. Since the OSDI questionnaire does not differentiate between aqueous-deficient and evaporative DE disease, the MGDQ, which is more specific to MGD, was also included [39].

The KEPAQ comprises 16 items divided into two sub-scales. The first part includes seven questions assessing the emotional impact of the disease (KEPAQ-E), while the second sub-scale consists of nine questions addressing the functional impairment associated with the ectasia (KEPAQ-F). Each question employs a Likert response format, with scores assigned as follows: “Not at all” = 3; “A little” = 2; “Quite a Bit” = 1; “A Lot” = 0; and “Not Applicable” if the question is irrelevant. The sum of scores across all questions is converted to an individual score based on Rasch analysis [27]. A higher score indicates less disability related to the disease.

### 2.3. Statistical Analysis

Using G-Power software (ver. 3.1.9.7; Heinrich-Heine Universitat Dusseldorf, Düsseldorf, Germany) [42], the minimum sample size required for assessing correlations that are not normally distributed, for a power (1 − β) of 80%, error probability of 5% (α error 0.05), and an effect size (d) of 0.40, was identified as 44 participants. Diagnostic outcome measures for DE included the Ocular Surface Disease Index (OSDI) score and non-invasive BUT scores. Diagnostic outcome measures for MGD included the DE measures and meibography scores, non-invasive tear break. Non-DE clinical measures included high-contrast distance Snellen decimal visual acuity, spherical refraction, cylindrical refraction, keratometry, and Belin outcomes. DE clinical measures included Schirmer test scores, meibum expressibility, meibum quality, tear meniscus height, corneal staining, conjunctival staining, and MGD questionnaire scores. 

Outcome measures were recorded in an Excel spreadsheet for analysis. KEPAQ-E and KEPAQ-F scores were calculated and converted using Rasch analysis as described above.

Statistical calculations were carried out using SPSS version 26 (SPSS Inc., Chicago, IL, USA). The normality of each outcome measure was assessed using the Shapiro–Wilks test. Descriptive statistics were applied. Given that the data were normally distributed, associations between DE outcome measures and questionnaire scores were examined using Pearson correlations. Differences between sub-groups were compared using *t*-tests.

As patient-reported outcome measures reflect the overall visual experience, they inherently apply to both eyes. However, previous studies have shown stronger associations between quality of life scores and clinical findings in the more advanced eye [43], with worse visual acuity contributing more significantly to emotional quality of life [27]. Therefore, the current analysis was based on the eye with the higher average keratoconus index [44,45]. The data were analyzed for the full sample, and also for specific sub-groups: with vs. without DE diagnosis, and with vs. without MGD diagnosis.

Pearson’s bivariate correlation tests examined associations among four specific study variables: OSDI and MGD symptom scores and emotional and functional KEPAQ scores. To estimate the simultaneous effects of dry eye symptom scores (OSDI and MGD) on KEPAQ scores (emotional and functional), while controlling for shared variables among the predictors and criterion variables, a multivariate path analysis model with AMOS (Version 29, [46]) was applied using the maximum-likelihood method.

## 3. Results

Clinical outcome measures of the 45 participants with KC that were included in the study (N = 45 eyes, mean age: 45.1 ± 13.2, range: 20 to 69 years, 25 males) as well as the dry eye (N = 22, 49%)/non-dry eye (N = 23, 51%) and MGD (N = 15, 33%)/non-MGD (N = 30, 67%) sub-groups, are summarized in Table 1. All participants had bi-lateral keratoconus.

The Belin grading outcomes of the 45 participants and the respective sub-groups are displayed in Appendix A, Table A1. The MGD sub-group significantly differed from the non-MGD sub-group in parameters C (thinnest pachymetry, *p* = 0.04) and D (visual acuity, *p* = 0.01). Specifically, in the MGD sub-group only 4 eyes (33%) had Belin C grade 0 and 5 eyes (42%) had Belin C grade 2, compared with 18 eyes (64%) and 3 eyes (11%) in the non-MGD group, respectively. Additionally, in the MGD sub-group, 6 eyes (40%) had Belin D grade 1, 5 eyes (33%) had Belin D grade 2, and 3 eyes (20%) had Belin D grade 3, compared with 12 eyes (41%), 3 eyes (10%), and one eye (3%) in the non-MGD sub-group, respectively.

**Dry eye vs. non-dry eye:** KEPAQ-E scores were significantly negatively correlated with Belin outcome B both dry eye and non-dry eye sub-groups.

KEPAQ-E scores were significantly negatively correlated with Belin outcome C (thinnest pachymetry) for the non-dry eye sub-group. Loss of MGs in the lower eyelids and MGD questionnaire scores were significantly correlated with KEPAQ-E scores for the dry eye sub-group. KEPAQ-F scores were significantly negatively correlated with Belin outcome B and the MGD questionnaire scores for both the dry eye and non-dry eye sub-groups. KEPAQ-F scores were significantly negatively correlated with Belin outcomes A and D for the non-dry eye sub-group. They were also negatively correlated with loss of MGs in the lower eyelids in the dry eye group.

**MGD vs. non-MGD:** KEPAQ-E and KEPAQ-F scores were significantly negatively correlated with Belin outcome A (anterior radius of curvature), Belin outcome C, Belin outcome D (distance visual acuity), and MGD questionnaire score for the non-MGD group.

The mean KEPAQ-E and KEPAQ-F scores for all participants (Table 2) were 2.85 ± 3.02 and 1.74 ± 3.04 Logit, respectively. KEPAQ scores were compared across sub-groups using ANCOVA. Each Belin outcome parameter was initially tested as a covariate to control for disease severity; however, given the high intercorrelation among all Belin indices (indicating multicollinearity) and the observation that results remained consistent across models, Belin D was selected as the representative variable for adjustment. A significant difference in KEPAQ-E scores was found between MGD and non-MGD sub-groups (*p* = 0.03).

**Entire cohort:** As seen in Table 3, KEPAQ-E and KEPAQ-F scores were significantly negatively correlated with all Belin outcome measures A-D, as well as the MGD questionnaire scores for all participants with keratoconus.

**Figure 1:**
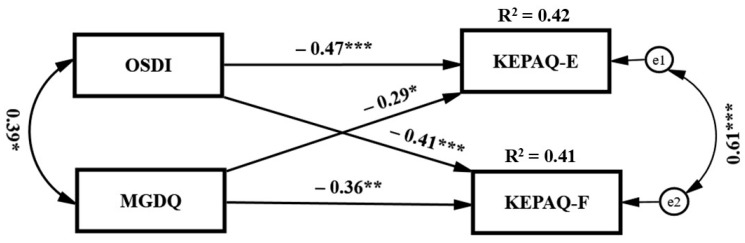
Path analysis for the effects of OSDI and MGDQ on KEPAQ scales controlling for shared variance among the Ocular Surface Disease Index (OSDI) scores and the Meibomian Gland Dysfunction Questionnaire (MGDQ) scores, as well as for the shared variance among the Emotional and Functional Items in the Keratoconus End-Points Assessment Questionnaire (KEPAQ). Note. * *p*< 0.05; ** *p*< 0.01, *** *p*< 0.001.

Univariate analyses indicated that Mean KEPAQ-E and KEPAQ-F scores were significantly correlated with both OSDI and MGDQ scores (rs range: −0.48 to −0.59, *p* < 0.001). However, there is a relationship between OSDI and MGDQ scores (r = 0.39, *p* < 0.01), as MGD is a type of dry eye. Further, KEPAQ-E and KEPAQ-F are parameters within the same questionnaire and are therefore related to each other (r = 0.77, *p* < 0.001). Thus, to evaluate the simultaneous effects of DE symptom scores on the emotional and functional KEPAQ scores, while controlling for the shared variables among the predictors and criterion variables, a conservative path analysis was applied, as presented in Figure 1.

The multivariate analysis indicated that when controlling for the shared variance among OSDI and MGDQ—the predictors (r = 0.39, t = 2.43, *p* < 0.015) and for the shared variance among KEPAQ-E and KEPAQ-F scores—the outcomes (r = 0.61, t = 3.46, *p* < 0.0001), both OSDI and MGDQ were significantly associated with low emotional (OSDI: β = −0.47, t = −3.79, *p* < 0.0001; MGDQ: β = −0.29, t = −2.299, *p* < 0.05) and low functional KEPAQ scores (OSDI: β = −0.41, t = −3.24, *p* < 0.001; MGDQ: β = −0.36, t = −2.82, *p* < 0.01). Pairwise Parameter Comparisons indicated that while the magnitude of the effect of OSDI on KEPAQ-E was almost significantly stronger than the magnitude of the effect of MGDQ on KEPAQ-E (t = 1.92, *p* < 0.05 *two tailed*), the magnitude of the effect of OSDI on KEPAQ-F was significantly stronger than the magnitude of the effect of MGDQ on KEPAQ-F (t = 2.47, *p* < 0.05). Moreover, Pairwise Parameter Comparisons indicated that the magnitude of the effects of OSDI on each of the KEPAQ scales (−0.47 vs. −0.41) as well as the magnitude of the effects of MGDQ on these scales (−0.29 vs. −0.36) were not significantly different.

This conservative model that controlled for the shared variance among the predictors and the shared variable among the outcomes significantly explained 42% of the variance in KEPAQ-E and 41% of the variance in KEPAQ-F scores.

## 4. Discussion

This study examined whether functional and emotional quality of life, as assessed by the KEPAQ, are influenced by subjective symptoms and clinical signs of DE compared to those specifically associated with MGD, in patients with KC. The findings reveal significant negative correlations between Belin outcome parameters and KEPAQ questionnaire scores across the entire cohort. These results align with Balparda et al. [27], who reported significant inverse correlations between Belin criteria A, B, and D and both KEPAQ subscales in their cohort of 114 KC patients. The reported significant associations do not distinguish if the reduced quality of life is specifically attributable to the presence of dry eye disease or meibomian gland dysfunction. The finding of significant KEPAQ-E scores between the MGD and non-MGD sub-groups after adjusting for Belin D suggests that MGD may have an independent adverse effect on emotional aspects of quality of life in keratoconus patients, beyond the impact of keratoconus itself. The findings highlight the need for clinicians to routinely assess and manage dry eye symptoms in keratoconus patients to improve their overall well-being and quality of life. Implementing targeted interventions, such as lubricating eye drops or meibomian gland therapy, could significantly alleviate the emotional and functional burden experienced by these patients.

The present study builds upon previous research by specifically analyzing the associations between KEPAQ scores and clinical measures within dry-eye and MGD sub-groups. A significant relationship was observed for the dry eye sub-group, indicating that reduced quality of life in keratoconus may, at least in part, be attributed to the presence and severity of dry eye disease. Clinical measures that demonstrate significant associations in the dry eye or MGD groups without significant associations in the non-dry eye and non-MGD groups can be considered clinical indicators (such as loss of meibomian glands in the lower eyelids) for reduced quality of life. The emotional and functional KEPAQ scores of the non-dry eye and non-MGD sub-groups were predominantly associated with objective structural and refractive parameters of keratoconus, suggesting that functional and emotional aspects are primarily driven by corneal pathology. Conversely, in patients with keratoconus who were diagnosed with dry eye, drivers of functional and emotional quality of life were more complex. While still influenced by keratoconus parameters, the scores were also significantly correlated with meibomian gland loss in the lower eyelids. This finding underscores the multifactorial nature of reduced quality of life in patients with keratoconus, with dry eye contributing more significantly to this impairment.

Furthermore, to investigate the relationship between symptoms specific to dry eye (OSDI) and symptoms specific to MGD (MGD questionnaire) and the emotional and functional quality of life in keratoconus, KEPAQ scores were taken as outcome measures, and evaluated through correlation and path analysis. Belin criteria D, best corrected visual acuity, was significantly correlated with KEPAQ-F, indicating that visual acuity impacts functional aspects of quality of life. Visual acuity may not ensure good quality of vision and quality of life, especially in patients with KC [20]. Further, patients with keratoconus whose visual acuity is normal, may still report impaired quality of life [47]. Thus, the impact of the highly subjective visual alteration due to keratoconus is more appropriately evaluated using subjective questionnaires such as KEPAQ [27].

There was a significant difference in the distance visual acuity of the MGD and non-MGD sub-groups, which is approximately two lines on the Snellen acuity chart. This difference is considered clinically meaningful [48]. This could be due to the fact that the two groups also significantly differed in their cylindrical refractive component (*p* = 0.02) by a mean difference of more than 1.00 DC, and the participants were asked to attend the study visit with their spectacle rather than contact lens correction.

Dry eye disease and meibomian gland dysfunction are characterized by tear film instability. The tear film breakup creates an irregular ocular surface, which disrupts smooth vision [49], introduces anterior corneal higher order aberrations [50], fluctuations in vision, and image blur impairing visual quality [51]. These visual disturbances negatively affect quality of life and psychological well-being [52]. They also impair occupational functioning by necessitating frequent blinking or artificial tear use, thereby reducing workplace productivity [49].

KEPAQ-E and KEPAQ-F scores were significantly correlated with both OSDI and MGD questionnaire (MGDQ) scores, with OSDI symptom scores demonstrating a more significant impact on both functional and emotional keratoconus quality of life indices compared with MGDQ scores.

In this study, out of 45 participants diagnosed with KC, 22 (49%) were diagnosed with DE, and 15 (33%) were diagnosed with MGD. This prevalence is lower than the 64% reported for the general population in the Northern West Bank of Palestine. The higher prevalence of DE in their study may be explained by the higher proportion of female participants (53% of their 769 participants compared with 44% in the present study), as DE is more common among women [3]. It may also be explained by the wider age range of their participants (18–90 years compared with 20–69 years in the present study), as DE is known to increase with age [3].

Our previous study [53] on a similar geographic cohort reported a prevalence of DE of 28% and a prevalence of MGD of 4% among normal young participants (aged 18–39). The higher prevalence of DE and MGD in the present study may be attributed to the KC [10,18] and the higher average age of the participants (mean age: 45, range: 20–69 years) [2,54].

A previous study that only examined symptoms among participants with KC based on OSDI scores ≥ 33 found that 90% reported symptoms [55]. Applying the same criterion in the present cohort resulted in 33% of participants reporting symptoms. That study [55] did not report the severity of KC, and it is possible that their cohort had more severe cases of KC, which are expected to be more symptomatic. Furthermore, their age range was between 15–87 years, whereas the present study’s range was 20–69 years. Thus, their cohort included older patients, which may also explain the increased symptoms.

Consistent with the present findings, Martínez-Pérez et al. [56] reported a 56% prevalence of DE among their KC cohort (mean age: 35, range: 13–66 years), compared with 32% in their control group (mean age: 37, range: 15–63 years). The similarities in the prevalence of DE between the two studies can be attributed to parallels in the characteristics of the study cohorts. However, they observed a 49% prevalence of MGD among their KC participants (compared to 29% in controls), which is higher than our reported prevalence of 33%. This discrepancy may be due to the fact that their MGD diagnosis relied on functionality of the glands (meibomian gland expressibility), whereas the present study was based on the morphology of the glands (meibography).

Surprisingly, the MGD questionnaire score was significantly different only for the DE vs. non-DE groups and approached significance in the MGD vs. non-MGD groups. In both cases, however, the mean difference in the scores was approximately 1 point (median: 2) indicating that they only differed in one of the responses, which is not clinically meaningful.

The main goal of this study was to determine whether subjective symptoms and clinical signs of DE and MGD differentially contribute to emotional and functional quality of life impairments in KC patients. Statistical modeling was applied to assess how these factors correlate with KEPAQ-E and KEPAQ-F scores. A previous study [27] examined correlations between Belin ABCD classification and emotional and functional KEPAQ scores reported mean scores of 2.33 ± 3.40 and 1.85 ± 3.61 Logit, respectively. This emotional score is approximately 0.5 Logit lower, and the functional score is approximately 0.1 Logit higher than in the present study, indicating that their cohort had more emotional compromise and less functional compromise compared to the cohort of the present study. Their research reported that criteria A and D from the Belin classification were significantly negatively associated with KEPAQ-E and KEPAQ-F scores. Their study also did not examine clinical measures of dry eye, which is a novel aspect of the present investigation.

This study offers valuable insights into the relationship between dry eye (DE), meibomian gland dysfunction (MGD), and quality of life in keratoconus (KC) patients. However, several limitations should be acknowledged. First, while validated questionnaires were utilized, self-reported measures may introduce subjective bias. Nevertheless, the present study proposed a relatively conservative model that accounted for the strong and significant associations related to both self-reported measures and within-subject variance, demonstrating significant effects of reported symptoms of dry eye on subjective quality of life in patients with keratoconus. Patient-reported outcome measures provide essential insight into patients’ symptoms, functioning, and satisfaction. This input is increasingly seen as complementary to objective clinical assessments, and essential in improving patient care [57]. Second, environmental and lifestyle factors influencing DE symptoms, such as screen time, humidity, and habits, were not controlled for, which may have influenced the outcomes. Additionally, the respondents were not specifically asked about their environmental and lifestyle factors, which could have contributed to the outcomes. Third, the wide range of visual acuity of the participants may be a confounder as visual acuity affects quality of life. The present cohort included 12 KC patients with visual acuity < 0.5 (Snellen decimal, equivalent to less than 6/12) which is defined as having a visual impairment [58]. The KEPAQ-E and KEPAQ-F scores of these 12 participants were 2.36 ± 3.57 and 0.42 ± 3.32, respectively. When compared to the scores of the 33 participants with visual acuity better than 0.5 (2.93 ± 2.81 and 2.07 ± 2.75, respectively) it is apparent that the emotional and functional quality of life is influenced by the visual acuity. Finally, given the presence of relationships among the clinical outcome measures and their associations with quality of life indices, we recommend that future studies include larger datasets to support multivariate regression analyses. This approach will enable the examination of the combined predictive value of clinical outcome measures on quality of life indices, while accounting for the shared variance among predictors. Such modeling would allow for an assessment of the unique contribution of each variable and refining our understanding of which specific clinical outcomes are most impactful in predicting quality of life of patients with keratoconus.

## 5. Conclusions

Emotional and functional measures of quality of life in patients with keratoconus were significantly associated with dry eye and meibomian gland dysfunction symptoms. The impact of dry eye symptom scores on both functional and emotional keratoconus quality of life indices was significantly greater than that of the meibomian gland dysfunction symptoms scores. Among keratoconus patients with diagnosed dry eye quality of life scores were also significantly correlated with loss of meibomian glands in the lower eyelids. These findings underscore the importance of including dry eye assessments in the management of keratoconus patients to improve patient-reported outcomes. In conclusion, our study demonstrates a significant relationship between dry eye and meibomian gland dysfunction symptoms and the emotional and functional quality of life in keratoconus patients. Emphasizing the importance of dry eye assessments in the management of keratoconus, our findings aim to improve patient-reported outcomes by identifying and addressing these contributing factors.

## Figures and Tables

**Table 1:** Clinical outcome measures of the participants with keratoconus, dry eye (DE) and non-dry eye, meibomian gland dysfunction (MGD), and non-MGD sub-groups. The means (±standard deviations), medians, ranges, and first and third quartiles (Q1, Q3) for each outcome measure are detailed. *p*-values represent differences between sub-groups compared using *t*-tests.

Outcome Measure			Dry Eye (N = 22)	Non Dry Eye (N = 23)	*p*	MGD (N = 15)	Non-MGD (N = 30)	*p*	All (N = 45)
**Distance Visual Acuity** **(Snellen Decimal)**		**Mean ± SD** **Range**	0.61 ± 0.35 0.05–1.00	0.79 ± 0.27 0.10–1.00	0.07	0.50 ± 0.34 0.05–1.00	0.81 ± 0.26 0.10–1.00	0.003	0.70 ± 0.32 0.05–1.00
		**Median** **(Q1,Q3)**	0.75 (0.30, 0.90)	0.85 (0.70, 1.00)		0.40 (0.20, 0.90)	0.90 (0.70, 1.00)		0.80 (0.40, 1.00)
**Spherical Refraction** **(D)**		**Mean ± SD** **Range**	−1.55 ± 3.81 −10.25–6.00	−1.64 ± 2.91 −7.25–3.75	0.93	−1.57 ± 4.12 −10.25–6.00	−1.61 ± 2.96 −7.25–3.75	0.97	−1.59 ± 3.34 −10.25–6.00
		**Median** **(Q1,Q3)**	−0.75 (−4.25, 1.00)	−1.00 (−3.50, 0.75)		−0.75 (−4.25, 1.25)	−0.88 (−3.50, 0.75)		−0.75 (−4.00, 0.75)
**Cylindrical Refraction** **(D)**		**Mean ± SD** **Range**	−4.36 ± 3.34 −11.75–−0.75	−3.66 ± 4.65 −18.75–−0.25	0.12	−5.20 ± 3.52 −11.75–−0.75	−3.41 ± 4.19 −18.75–−0.25	0.02	−4.01 ± 4.03 −18.75–−0.25
		**Median** **(Q1,Q3)**	−3.50 (−6.75, −1.75)	−2.00 (−3.00, −1.25)		−4.50 (−7.75, −2.75)	−1.88 (−3.00, −1.25)		−2.50 (−4.75, −1.25)
**Keratometry** **(mm)**		**Mean ± SD** **Range**	7.56 ± 0.92 6.32–10.89	7.52 ± 0.57 6.32–8.47	0.32	7.56 ± 1.08 6.32–10.89	7.53 ± 0.55 6.32–8.55	0.29	7.54 ± 0.75 6.32–10.89
		**Median** **(Q1,Q3)**	7.44 (7.14, 7.71)	7.62 (7.26, 7.81)		7.44 (7.11, 7.71)	7.58 (7.36, 7.77)		7.57 (7.26, 7.76)
**NITBUT** **(sec)**		**Mean ± SD** **Range**	7.70 ± 4.78 1.70–17.00	10.84 ± 5.42 2.70–18.00		8.09 ± 4.88 1.70–17.00	9.91 ± 5.48 2.70–18.00		9.30 ± 5.30 1.70–18.00
		**Median** **(Q1,Q3)**	6.50 (4.20, 9.40)	9.80 (5.20, 17.00)		7.00 (4.20, 11.20)	8.50 (5.20, 17.00)		7.70 (5.20, 14.30)
**Schirmer test** **(mm)**		**Mean ± SD** **Range**	13.91 ± 10.54 2.00–35.00	11.87 ± 11.68 0.00–35.00	0.52	13.33 ± 9.48 2.00–30.00	12.63 ± 11.92 0.00–35.00	0.78	12.87 ± 11.06 0.00–35.00
		**Median** **(Q1,Q3)**	13.00 (3.00, 22.00)	8.00 (4.00, 20.00)		14.00 (3.00, 22.00)	8.00 (4.00, 22.00)		9.00 (4.00, 22.00)
**MG loss** **(0–100%)**	**Upper**	**Mean ± SD** **Range**	27.11 ± 19.16 8.33–93.47	28.12 ± 13.38 5.50–59.67		32.96 ± 20.07 10.23–93.47	24.97 ± 13.49 5.50–59.67		27.63 ± 16.19 5.50–93.47
		**Median** **(Q1,Q3)**	23.88 (14.87, 31.75)	28.55 (18.03, 34.30)		28.78 (23.27, 34.03)	24.97 (15.05, 33.60)		27.07 (16.67, 34.03)
	**Lower**	**Mean ± SD** **Range**	30.17 ± 19.44 8.13–70.20	26.67 ± 14.73 7.17–65.63		37.11 ± 18.93 8.13–70.20	23.71 ± 14.24 7.17–65.63		28.38 ± 17.07 7.17–70.20
		**Median** **(Q1,Q3)**	25.57 (13.03, 42.00)	21.92 (16.33, 39.77)		29.27 (25.13, 56.33)	18.38 (13.95, 31.92)		24.50 (14.67, 39.94)
**OSDI score** **(0–100)**		**Mean ± SD** **Range**	45.57 ± 23.00 16.67–100.00	8.19 ± 14.38 0.00–70.83		47.67 ± 23.12 18.75–100.00	15.86 ± 21.79 0.00–81.25		26.46 ± 26.70 0.00–100.00
		**Median** **(Q1,Q3)**	45.74 (22.92, 59.37)	6.25 (0.00, 10.42)		47.73 (22.92, 60.00)	8.71 (2.08, 16.67)		16.67 (6.25, 47.73)
**MGD symptoms questionnaire** **(0–11)**		**Mean ± SD** **Range**	4.14 ± 2.38 0.00–8.00	2.57 ± 1.75 0.00–7.00	0.02	4.33 ± 2.53 0.00–8.00	2.83 ± 1.88 0.00–7.00	0.05	3.33 ± 2.21 0.00–8.00
		**Median** **(Q1,Q3)**	4.00 (2.00, 6.00)	2.00 (1.00, 4.00)		4.00 (2.00, 6.00)	2.00 (1.00, 4.00)		3.00 (1.00, 5.00)

**Table 2:** Keratoconus end-points assessment questionnaire (KEPAQ) responses. *p*-values represent differences between sub-groups adjusted for Belin D using ANCOVA. Belin D was used to account for disease severity due to high intercorrelation among Belin parameters.

Outcome Measure		Dry Eye (N = 22)	Non-Dry Eye (N = 23)	*p*	MGD (N = 15)	Non-MGD (N = 30)	*p*	All (N = 45)
**KEPAQ-E**	**Mean ± SD** **Range**	1.84 ± 2.72 −5.47–6.40	3.82 ± 3.03 −2.64–6.40	0.05	1.31 ± 2.69 −5.47–4.89	3.62 ± 2.91 −2.64–6.40	0.03	2.85 ± 3.02 −5.47–6.40
	**Median** **(Q1,Q3)**	2.22 (0.14, 3.72)	4.89 (1.26, 6.40)		2.22 (−0.09, 3.72)	3.88 (1.26, 6.40)		2.87 (0.55, 6.40)
**KEPAQ-F**	**Mean ± SD** **Range**	0.85 ± 2.60 −2.98–6.97	2.58 ± 3.25 −5.43–6.97	0.17	0.23 ± 2.43 −2.98–5.49	2.49 ± 3.07 −5.43–6.97	0.09	1.74 ± 3.04 −5.43–6.97
	**Median** **(Q1,Q3)**	−0.02 (−0.58, 2.12)	2.47 (0.54, 5.49)		−0.52 (−0.97, 2.00)	2.00 (0.47, 5.49)		1.21 (−0.40, 4.41)

**Table 3:** Correlation between Belin ABCD criteria, clinical outcome measures, subjective dry eye questionnaires, and the scores of both sub-scales of the KEPAQ questionnaire.

		KEPAQ-E R (*p*)					KEPAQ-F R (*p*)				
Variable		Dry Eye (N = 22)	Non-Dry Eye (N = 23)	MGD (N = 15)	Non-MGD (N = 30)	All (N = 45)	Dry Eye (N = 22)	Non-Dry Eye (N = 23)	MGD (N = 15)	Non-MGD (N = 30)	All (N = 45)
**A**		−0.36 (0.13)	−0.41 (0.07)	0.26 (0.35)	−0.50 (0.01)	−0.43 (0.006)	−0.29 (0.22)	−0.51 (0.02)	−0.18 (0.52)	−0.50 (0.01)	−0.35 (0.03)
**B**		−0.48 (0.04)	−0.45 (0.04)	−0.39 (0.16)	−0.34 (0.09)	−0.34 (0.03)	−0.55 (0.01)	−0.49 (0.03)	−0.39 (0.15)	−0.36 (0.08)	−0.32 (0.04)
**C**		−0.13 (0.60)	−0.45 (0.04)	−0.15 (0.59)	−0.62 (0.001)	−0.47 (0.002)	−0.20 (0.41)	−0.38 (0.09)	−0.15 (0.59)	−0.48 (0.02)	−0.37 (0.02)
**D**		−0.04 (0.87)	−0.06 (0.79)	−0.14 (0.60)	−0.46 (0.02)	−0.37 (0.01)	−0.05 (0.84)	−0.49 (0.02)	−0.34 (0.19)	−0.51 (0.007)	−0.44 (0.003)
**Schirmer test**		−0.42 (0.05)	0.11 (0.63)	−0.05 (0.86)	−0.11 (0.57)	−0.06 (0.70)	−0.17 (0.44)	0.18 (0.42)	−0.18 (0.50)	−0.05 (0.82)	−0.01 (0.93)
**MG loss**	**Upper**	−0.38 (0.10)	−0.26 (0.25)			−0.17 (0.29)	−0.15 (0.52)	−0.08 (0.71)			−0.11 (0.49)
	**Lower**	−0.44 (0.04)	−0.10 (0.68)			−0.06 (0.69)	−0.44 (0.04)	0.10 (0.66)			−0.20 (0.20)
**Meibum Expressibility**		−0.25 (0.27)	−0.33 (0.15)	−0.28 (0.34)	−0.25 (0.21)	−0.24 (0.13)	−0.22 (0.33)	−0.11 (0.65)	−0.42 (0.14)	0.05 (0.79)	−0.09 (0.57)
**Meibum Quality**		−0.10 (0.66)	−0.24 (0.30)	−0.11 (0.70)	−0.21 (0.29)	−0.16 (0.32)	−0.03 (0.91)	−0.16 (0.50)	−0.06 (0.85)	−0.07 (0.71)	−0.07 (0.65)
**T** **ear Meniscus Height**		0.31 (0.18)	−0.38 (0.07)	0.26 (0.37)	−0.24 (0.21)	−0.12 (0.44)	−0.08 (0.72)	−0.25 (0.25)	−0.08 (0.80)	−0.19 (0.31)	−0.18 (0.24)
**Corneal Staining**		−0.17 (0.46)	0.11 (0.63)	0.09 (0.76)	−0.02 (0.92)	−0.09 (0.57)	−0.22 (0.34)	−0.11 (0.60)	−0.17 (0.54)	−0.16 (0.39)	−0.21 (0.17)
**Conjunctival Staining**		0.20 (0.37)	0.00 (1.00)	0.10 (0.73)	0.14 (0.45)	0.10 (0.51)	0.00 (1.00)	−0.23 (0.28)	−0.006 (0.98)	−0.08 (0.66)	−0.08 (0.62)
**MGD questionnaire score**		−0.50 (0.02)	−0.32 (0.14)	−0.14 (0.59)	−0.47 (0.01)	−0.44 (0.003)	−0.53 (0.01)	−0.43 (0.04)	−0.22 (0.40)	−0.59 (0.001)	−0.56 (<0.001)

MG = Meibomian gland, MGD = meibomian gland dysfunction.

## Data Availability

The data presented in this study are available on request from the corresponding authors.

## Appendix A

**Table A1:** Distribution of Belin outcome scores (ranging from 0–4) for all participants, as well as dry eye and non-dry eye sub-groups, and meibomian gland dysfunction (MGD) and non-MGD sub-groups are described in numbers and percentages.

Outcome Measure	Stage	Dry Eye (N = 22)	Non-Dry Eye (N = 23)	*p*	MGD (N = 15)	Non-MGD (N = 30)	*p*	All (N = 45)
Belin A	0	11 (58%)	15 (71%)	0.70	5 (42%)	21 (75%)	0.09	26 (65%)
	1	1 (5.3%)	1 (4.8%)		1 (8.3%)	1 (3.6%)		2 (5.0%)
	2	4 (21%)	3 (14%)		3 (25%)	4 (14%)		7 (18%)
	3	0 (0%)	1 (4.8%)		0 (0%)	1 (3.6%)		1 (2.5%)
	4	3 (16%)	1 (4.8%)		3 (25%)	1 (3.6%)		4 (10%)
Belin B	0	13 (68%)	13 (62%)	0.33	6 (50%)	20 (71%)	0.10	26 (65%)
	1	0 (0%)	1 (4.8%)		0 (0%)	1 (3.6%)		1 (2.5%)
	2	1 (5.3%)	5 (24%)		1 (8.3%)	5 (18%)		6 (15%)
	3	2 (11%)	1 (4.8%)		2 (17%)	1 (3.6%)		3 (7.5%)
	4	3 (16%)	1 (4.8%)		3 (25%)	1 (3.6%)		4 (10%)
Belin C	0	10 (53%)	12 (57%)	0.69	4 (33%)	18 (64%)	0.04	22 (55%)
	1	2 (11%)	4 (19%)		2 (17%)	4 (14%)		6 (15%)
	2	5 (26%)	3 (14%)		5 (42%)	3 (11%)		8 (20%)
	3	1 (5.3%)	2 (9.5%)		0 (0%)	3 (11%)		3 (7.5%)
	4	1 (5.3%)	10 (45%)		1 (8.3%)	0 (0%)		1 (2.5%)
Belin D	0	4 (18%)	9 (41%)	0.06	1 (6.7%)	13 (45%)	0.01	14 (32%)
	1	9 (41%)	2 (9.1%)		6 (40%)	12 (41%)		18 (41%)
	2	6 (27%)	1 (4.5%)		5 (33%)	3 (10%)		8 (18%)
	3	3 (14%)	10 (45%)		3 (20%)	1 (3.4%)		4 (9.1%)
	4	0 (0.0%)	0 (0.0%)		0 (0%)	0 (0.0%)		0 (0.0%)
